# Evaluation of the Involvement of Pharmacists in Diabetes Self-Care: A Review From the Economic Perspective

**DOI:** 10.3389/fpubh.2018.00244

**Published:** 2018-09-03

**Authors:** Shazia Q. Jamshed, Mohammand J. Siddiqui, Bareera Rana, Akshaya S. Bhagavathula

**Affiliations:** ^1^Pharmacy Practice, International Islamic University Malaysia, Selayang, Malaysia; ^2^University of Veterinary and Animal Sciences, Lahore, Pakistan; ^3^Department of Internal Medicine, College of Medicine and Health Sciences, United Arab Emirates University, Al Ain, United Arab Emirates

**Keywords:** pharmacoeconomic analysis, diabetes self-care, pharmacist involvement, cost-effectiveness analysis, cost of utilization, cost of illness, cost of minimization, cost of benefit

## Abstract

**Objectives:** To analyze the studies encompassing the involvement of pharmacists in diabetes self-care.

**Method:** We reviewed studies conducted from 2005 to 2017 on the involvement of pharmacists in diabetes self-care. The keywords mainly used in this search are pharmacoeconomic analysis, diabetes self-care, pharmacist involvement,cost-effectiveness analysis, cost of utilization, cost of illness, cost of minimization and cost-benefit analysis. PubMed, Science Direct, Springer Link and Medline searched for the relevant studies. These databases searched for full text articles ranging from 2007 to 2017. We tried to limit the search with the inclusion of studies having any sort of pharmacoeconomically relevant component.

**Key Findings:** Cost of illness varied among the countries in managing diabetes mellitus, and the cost of managing diabetes complications were twice the cost of management of diabetes. Continuous involvement of the pharmacist in primary health care is a cost-effective strategy and pronounced to be essential for helping diabetes patient in controlling and managing their disease. Implementation of diabetes self-care by pharmacists such as lifestyle intervention rendered improved quality of life of patient without any increase in health care cost. Self-care management generates intensive blood glucose control and improved quality of life.

**Conclusions:** Implementation of diabetic self-care intervention including intensive lifestyle intervention, education, self-monitoring of blood glucose and adherence toward medication-initiated reduction in the overall healthcare cost of diabetic patients compared to patients relying on only any one of the interventions. Impact of diabetes self-care intervention by pharmacist reported to significantly reduce the HbA1C levels of diabetic patients along with the reduction of yearly healthcare cost. This review showed that pharmacist involvement in diabetes self-care interventions prove to be cost-effective and can significantly affect the condition of the diabetic patients and reduces the risk of complications.

## Introduction

Diabetes, a metabolic disorder with rapid emerging epidemic, accounts for a total of 387 million global population living with diabetes till 2014 (1). It is associated with substantial indisposition toward other illnesses and a major risk factor for cardiovascular disease, stroke, and renal complications (1). According to the National Health and Morbidity Survey (NHMS) 2011, “the prevalence of diabetes in Malaysia had amplified relatively from 15.2% (2011) to current 17.5%, compared to the relative increase of 31.0% between 2006 (prevalence: 11.6%) and 2011” (2). Diabetes cases also increase sequentially with age and are at its peak in the age group of 70–74 years (2). The intensifying drift in diabetes is distressing because the disease generates undesirable impacts on patients, their families and caregivers and society, both humanistically and financially owing to significant cost involved in diabetes care (1).

Diabetic patients generally handle their daily activities independently (3). Self-care management is important to halt the progression of diabetes and its complications as the goal of treatment is simply to prevent microvascular and macrovascular complications (4, 5). However, this proves to be difficult as the key to effective management lies in consistent cooperation of the patient. Generally, complications of diabetes manifest when there is inappropriate compliance to self-care recommendations which stems from poor self-discipline and education followed by lack of support from family members (6). Thus, it is important for the patients to realize that their self-confidence and self-discipline promote better self-care and overall improved health outcomes (7, 8). Self-care components comprises of self-monitoring of blood glucose (SMBG), diet control, optimum physical exercise, adherence to medication/s and proper foot and eye care (6).

Self-monitoring of blood glucose (SMBG) means that the initiative to regularly check and record blood glucose through glucometer must be with the patient. This continuous monitoring is expected to alert the patient of any discrepancies in the level and prompt to take appropriate measures. However, this vital component of self-care is generally missing in diabetic patients (6). In this Ethiopian study nearly half of study subjects checked their blood glucose level only during follow up visits (every 3–4 months) or when they feel ill (6). This emphasizes that the understanding and motivation of patients about SMBG is crucial and the patient needs to be highly motivated for regular checking of blood glucose. The mere act of blood glucose monitoring will be insignificant if the patient is not motivated to implement it on a regular basis and unable to comprehend the correct measure of self-checking (9, 10).

Adherence to dietary control and diet patterns are significant parameters in improving diabetes control. According to Tol et al. (3) diet control adherence and exercise are significantly associated with glycemic control. Among the self-care practices, dietary control was considered the most essential component as majority avoid ingestion of table sugars, sweet drink and foods (6). Contrary to diet control, physical exercise showed a negative result. The statistics from National Health and Morbidity Survey in 2015 showed that the level of physical activity gradually decreased with increasing age and apparently seen mostly in elderly. Lack of motivation, lack of interest, busy work schedule and patients' perspectives and lack of awareness regarding the benefits of exercise in diabetes seem to be implicated in neglecting proper self-care (6).

Self-care component of adherence toward medication can be defined as the proportion of medication taken and also defined as the average change of the amount of missed medication within the last seven days (11). Adherence to medication is important because it is one of the ways to prevent any possible complications in diabetic patients (12). In a systematic review of randomized controlled trials, significant changes in blood pressure and blood glucose levels were found in the intervention groups receiving pharmaceutical care in the study. Pharmacist also plays an important role in this self-care component as the involvement of a pharmacist contributes to an improvement of knowledge and self-care activities (11).

A major cause of morbidity and disability for people with diabetes are foot ulcers and amputations. It is important for having early recognition and management of independent risk factors for ulcers and amputations as it can prevent or delay the onset of adverse outcomes. There were few recommendations that has been suggested by Mayfield and colleagues (13). People with evidence of increased plantar pressure, such as erythema, warmth, and callus, should use footwear that cushions and redistributes the pressure. Other than that, people who are with extreme bony deformities that cannot be accommodated with the commercial therapeutic footwear, may need custom-molded shoes.

## Objective

To review the involvement of pharmacists in diabetes self-care from an economic perspective.

### Method

A literature search was performed from September 2017 to November 2017 to identify published studies related to the analysis of the involvement of pharmacists in diabetic self-care. Any study related to the economical perspective was included in the review. PubMed, Science Direct, Springer Link and Medline were used to search and retrieve the articles. The terms and keywords applied were “pharmacist involvement,” “diabetic self-care,” “cost-effectiveness,” “cost-minimization,” “cost-utilization,” “cost-benefit,” “cost-illness” and combinations of these terms. We reviewed the evidence from different types of studies such as meta-analysis study, systematic review, longitudinal qualitative study, descriptive cross-sectional study and randomized control trial. The search was limited to articles published in English within 12 years' time frame; i.e., between 2005 and 2017. Overall, a total of 4 articles were retrieved which encompass the contribution of pharmacists from the economical perspective.

### Cost effectiveness analysis

A 66-month program called The Patient, Pharmacists Partnership (P^3^ Program) by Rodriguez de Bittner et al. (14) controlled the effectiveness regarding clinical outcome and cost saving of continuous pharmacist-delivered services in employed diabetic patients over 5 years period. Six self-insured employers offered this program to their employees and family members with type 1 or types two diabetes for 3.5 years. The effectiveness of this program regarding clinical outcome evaluated using percent changes in laboratory measures from baseline to the latest and recent follow up-value; where the primary clinical outcome changes were in HbA1c value while secondary clinical outcome changes were in LDL, systolic blood pressure (SBP) and diastolic blood pressure (DBP). For economic outcomes, the patients' cumulative medical, pharmacy, and total costs, including copayments, were compared for the 12 months before and after their baseline measures (14).

A significant decrease in HbA1c value within 6 months after the initiation of the program observed in patients whom initial value was out of standard range. The same goes with LDL value, SBP value and DBP value which showed significant decrease within 12 months in patients with out-of-range value initially. The effect of P3 Program toward therapeutic goal decreases over time which may be due to the patients become less sensitive to engage with the program over a period. However, this may be due to patient became overconfident in managing their diabetes alone leading to their gradual decrease in participation of the program (14).

Regarding economic outcome, the average per-patient all-cause medical care costs significantly declined by $1321 (26%, *P* < 0.001) and the decrease contributed a 33% decline in emergency department visits and hospital admissions in the year following enrolment in the program. Meanwhile, the reduction in all-cause medical care cost came together with an increase of $603 (17%, P $\frac{1}{4}$ 0.20) in prescription drug costs per patient per year to the employer. The difference between these two costs lead to the saving which averaged around $717 (8.3%) per patient per year in total annual health care costs to employers (P $\frac{1}{4}$ 0.32), unadjusted for changes in copayments for diabetes-related medications and supplies averaging $309 per patient; although waived for the patient but paid by the employer in the second year. After subtracting this figure from employer's estimated post-year pharmacy cost, annual prescription drug costs increased by $295 (8%, P $\frac{1}{4}$ 0.52) per patient, resulting in total annual health care costs decline of $1031 (12%, P $\frac{1}{4}$ 0.15) per patient (14).

In a study conducted by Simpson et al. (15), the authors concluded that adding pharmacists in primary healthcare to decrease cardiovascular risk in type 2 diabetes mellitus is a cost-effective strategy. During the 1-year program in five Canadian primary care clinics, 260 patients with type 2 diabetes mellitus were divided into a control group and intervention group. The intervention group had sessions with the pharmacist, who conducted medication history and physical examination and any issues with medication management recommended to be addressed with the patients during the follow-up session. Meanwhile, the control group received usual care without any intervention from the pharmacist. After one year, all patients were seen face to face to determine the effectiveness of the program (15). The primary outcome was a decrease in ≥10% in systolic blood pressure, while the secondary outcome was a reduction in 10-year cardiovascular risk using the UK Prospective Diabetes (UKPDS) Risk Engine. For cost-effectiveness analysis, the intervention considered cost-effective if; (1) it costs less (negative incremental cost) and more effective (positive incremental health effect); or (2) it cost more but more useful than comparator and society is willing to pay for the additional cost (15).

The result shows that the mean annual cost for the pharmacist intervention was estimated to be $226 (±$143) per patient. There was an overall cost saving of $190 for the intervention group, but the difference with the control group was statistically insignificant (95% CI –$1040, $668). The cost-effectiveness analysis shows that the pharmacist intervention is dominant compared to control group because it costs less (–$190) and more effective accounting for 66% of 10,000 bootstrap replications (15). When a societal cost-effectiveness threshold (society's willingness to pay for a reduction of 1% in cardiovascular risk) of $31,500 considered, the probability that the intervention would be cost-effective was 99%. Conclusively, a pharmacist intervention is cost-effective for reducing cardiovascular risks in type 2 the patients (15).

The research from Taylor and associates (16) on the economic evaluation of a community pharmacy-delivered disease state management (DSM) service for type 2 mainly focussed on developing, implementing, and evaluating a standardized disease state management program for diabetes in community pharmacy to boost and enhance patient health and lower health care costs. Taylor et al. (16) offered and executed specialized care for type 2 diabetes in DSM project as compared to standard care regarding the point of view of the healthcare sector. There were an intervention and control groups. The intervention group provided with the specialized service that included one initial visit with six follow-up visits, all of which took place over approximately 9 months whereas the control group was assessed at baseline and then again at 9 months only. The intervention group enjoyed the privileges such as being instructed about blood glucose monitoring and were provided with Medisense blood glucose monitors and a medication review by a trained pharmacist if the patient had issues related to their medications (16). Patients also advised about their lifestyle and self-care, and the downloaded blood glucose monitoring data were printed out and discussed at each visit. For the control group, during that 9-month period, patients received no further intervention but only the standard care they usually received. It was observed that for the intervention group, the total cost of providing specialized services including pharmacist time, medication review, prints out, phone calls with diabetes-related healthcare such as medicine costs and resources used was $A1821.12 per patient. On the other hand, for control group which did not have any specialized services but with only standard services of medications, the total cost was lower with value of $A1437.81 per patient (16).

Also, for the effectiveness, it was noted that the HbA1c levels were decreased by 0.46% from baseline for the intervention group whereas for the control group a reduction of only 0.03% was marked. Moreover, an outstanding decrease on participants with elevated HbA1c at follow-up in the intervention group, which seemingly not achieved in the control group. To get a 0.43% reduction in the HbA1c achieved by the specialized services, the cost calculated is $A383 per patient per 9 months (16).

### Cost benefit analysis

Cost-benefit analysis (CBA) in type 2 diabetes mellitus self-care management notably observed in Australia and Malaysia. The Malaysian study aimed to analyze the CBA for the program named diabetes self-management program, DSMP in short, by utilizing the payment card contingent valuation (CV) method (17). The Australian study known as SugarCare program only involved community pharmacies in three regions of New South Wales (18). The study aimed to collect evidence concerning the benefit of the optimized diabetes care given by the community pharmacies in comparison to the standard care, regarding patient preference and CBA (18). SugarCare compared the control and intervention groups concerning the overall diabetes care, information about medicines, diet and exercise, discussion about medications, diabetes, and blood glucose, measurement of blood glucose, management of medications or disease problems and encouragement and support to change lifestyles. Duration of this program was about 9 months. After that, the consent was obtained to recruit the SugarCare patients for the consumer preference survey and CBA study by using a payment card to assess the maximum WTP (18). The consumer preference survey consisted of satisfaction and preference surveys. The response on the satisfaction survey of each SugarCare component was recorded using the Likert scale of 1–5 scale, whereby 1 indicated for “not very satisfied” and five was for maximum satisfaction. The participants of the preference survey were asked to choose between the scenario in control group (Scenario A-standard care) and the situation in intervention group (Scenario B-enhanced care).

Additionally, the WTP scores stated on the payment card were AUD3.50, AUD10, AUD30, AUD60, and patient preference amount of WTP. The intervention group had to attend six follow-ups to the participating community pharmacies apart from the first and the final visits. On the contrary, the patients in the control group only participated in the first and last visits. At the beginning and end of the program, the HbA1c, lipids, blood pressure and BMI of the respondents recorded. Initially, 239 patients enlisted into SugarCare. However, only 143 qualified patients joined SugarCare. Upon the completion of the program, 78 participants gave the consent to involve in the CBA study, with only 75 respondents successfully finished the survey (18). Forty-five patients of the intervention group and 30 patients of control group significantly satisfied with SugarCare components namely information about diet and exercise, measurement of blood glucose, management of medications or diabetic problems, discussion of diabetes and blood sugar and encouragement and support to change lifestyles. Generally, 44 patients preferred Scenario B than Scenario A, though this finding was insignificant (18). The WTP for Scenario A was AUD3.50 whereas WTP of Scenario B amounted to AUD10 (18).

## Discussion

In the current review all the included interventional studies (14–16, 18) reflected that pharmacist patient program or collaboration had good economic outcomes and also advocated improved health outcomes in the long term. A positive noted was also observed for WTP values for their favored care.

The study Taylor et al. (16) found out that HbA1c levels decreased by 0.46% in the intervention group compared with a change of 0.03% in the control group after 9 months. Moreover, the study also mentioned that the yearly costs for intervention for each patient is calculated with $A383 in the first 9 months and it is probable to positively affect in a saving the healthcare system long term. Similarly, study by Rodriguez et al. (14) showed that within the period of 6 months of the P^3^ program, there was an impressive decrease in HbA1c value of the participant and the sum of the yearly health care costs to employers was found to lessen by $1031 per beneficiary. With reference to both studies (14, 16), Hb1Ac can be used as a marker to prove the effectiveness of diabetes disease program management as several studies has shown that sustaining HbA1c level below 7% leads to lowering of risk of complications of diabetes and diabetes-related death. Moreover, in an economic analysis done by (15) proposed that the involvement of pharmacists is considered cost effective measure for reducing cardiovascular risk in patients with Type 2 diabetes.

From the studies done, it can be deducted that there is mark reduction in the Hb1Ac reading with the involvement of the pharmacist. Therefore, based from our analysis it can be said pharmacist intervention in the diabetes self-care can be regarded as cost effective. Generally, there are some limitation from the studies above which is the study are only restricted to a 1-year time thus it can affect the long-term cost-effectiveness of adding pharmacists to primary care teams. Secondly, health utilization information was obtained from patient self-report, which may influence the reliability. Thirdly, in the study the participant are varied in term of mean Hb1Ac levels at baseline therefore it can affect the final interpretation of reduction in Hb1Ac.

Both studies for CBA used WTP method via payment card to measure benefit using the monetary value. WTP is used to measure both indirect and intangible benefits. WTP is measured by CV that includes hypothetical scenario and bidding vehicle. DSMP and SugarCare studies employed bidding vehicle by applying the payment card, only differed in the monetary unit. DSMP used Ringgit Malaysia (RM) whereas SugarCare benefit was measured by Australian dollars (AUD) of 2002 currency value.

The study in USM, Malaysia included the involvement of two pharmacists in its diabetes self-care program (17). The pharmacists might contribute to the program via the components namely the diabetes medications related problems and diabetic foot care management. The patients might benefit from the DSMP based on the life expectancy. It was believed that the program could extend about 5 years of the life span of being diabetic. In contrast, DSMP might elicit the life span of the diabetic patient for 7.5 years because it was expected that the diabetic patient might get ESRD at the age of 67.5 years old from the normal life expectancy of Malaysians, which is 75 years old (17).

In comparison, the net benefit of DSMP to prevent one case of ESRD among diabetic patients is greater than the net benefit of this program in overall diabetic clinical course. Overall, this program is economically beneficial because its benefit to cost ratio was greater than one, though only by 0.2 point. Significant higher WTP was observed among the richer, well-educated and longer diabetic state patients. Richer respondents might want to pay more for the program if they appreciated the benefits they would get from DSMP. Higher education patients might acknowledge the severity of diabetes mellitus complications and eager to prevent them from happening. Lastly, the diabetic patients that had been in diseased state for longer period probably were highly motivated to get better health after their participations in DSMP thus they wanted to spend at higher cost if DSMP would be continued in the future (17).

Conversely, SugarCare program focused on the engagement of community pharmacists in the diabetes care. Several eligible SugarCare patients withdrawn themselves from the consumer preference and WTP surveys, with 17 of them stated their reasons of study withdrawal. About 19 respondents favored SugarCare similarly with the visit to the diabetes specialist with significant value of 0.005 (18). There is insignificant difference in total satisfaction between the standard diabetes care and enhanced diabetes care. In contrast, Intervention group had shown prominent satisfaction in individual SugarCare component namely the management of diabetes medications and diabetes issues.

Meanwhile, overall WTP values, measured by median maximum WTP value was remarkably greater for Scenario B, which amounted to AUD10, as compared to AUD3.50 for Scenario A with *p*-value of < 0.03 based on the patients' care preference. However, the difference between the Intervention groups and the Control groups was not noticeable regarding this matter, since both of the groups could rate for either scenario. It was found that the WTP values were affected by the patients' income but this finding cannot be evaluated further due to the limited sample size in this study. The patients wanted to pay higher for Scenario B or optimized care because of the comprehensive care that was given by the pharmacists in those six follow-ups. Ironically, the Intervention and Control group could not be differentiated since there is possibility of having bias. For instance, most of the respondents in the Intervention group would opt to Scenario B because they had experienced the care provided and vice versa.

There were some weaknesses and limitations in the previous studies. The first study did not assess the CBA in more life-threatening complications such as diabetic ketoacidosis and hyperosmolar hyperglycemic nonketotic syndrome (HHNS). Moreover, the recruitment of the patients was difficult to be conducted. Inadequate study sample was observed because and most of the results obtained were derived from assumptions only. In addition, it was tricky to evaluate the exact number of complications that could be avoided owing to this program. The second study proposed the future study to take account of the incremental benefit assessment that is easily to be comprehended by the study subjects. The limitations detected were inadequate sample size and presence of bias in conducting that study. Limited sample size made the CBA study could not be generalized to the Australian population.

## Limitations of the study

Several limitations identified in the previous studies. Firstly, a small sample size of study subjects could not reflect other populations outside the study settings. Small sample size also might lead to inadequate data and therefore findings are sometimes not generalized. The researchers might have experience this limitation due to difficulty in recruiting the participants. Other than that, biases also observed in previous pharmacoeconomic analysis studies. These biases involved the choice of selecting the respondents and preference of treatments given to the patients. In CEA the participants required to self-report some of the data which might lead to the inaccuracy of data.

Besides, some of the studies included diabetes complications in the pharmacoeconomic analysis of their respective diabetes self-care programs. For instance, the CBA study was having difficulty in measuring the exact number of complications other than ESRD that could be prevented due to DSMP whereas the CEA study only evaluated cardiovascular diseases secondary to diabetes mellitus in one-year time. The cardiovascular diseases are chronic conditions which may take a longer time to progress. Due to the limited duration of the study, long-term CEA involving pharmacists in primary care teams was unclear. Therefore, the limitations mentioned beforehand contributed to the limitations of this study. Furthermore, the literature on diabetes self-care is in scarcity as compared to management of diabetes mellitus. Also, most of the research on this matter rarely involve pharmacists as a whole e and unlikely engagement of pharmacists in diabetes self-care, the involvement of other healthcare professionals and pharmaceutical companies well-documented. CEA studies are well-documented than other costs. Consequently, huge research gap between CEA and CUA literatures is observed.

## Recommendations

More future research should be directed to diabetes self-care because diabetes mellitus is a chronic disease which needs comprehensive management. Moreover, the involvement of pharmacists in diabetes self-care should be emphasized in future. Other costs such as COI, CMA, and CUA should also be the focus of pharmacoeconomic analysis rather than CEA to ensure adequate data in correlating cost and benefit of diabetes self-care.

## Conclusion

The overall costs of managing diabetic patients present with complication are more than twice compared to those without complications. The direct cost of illness can be reduced by implementation of diabetic self-care intervention including intensive lifestyle intervention, education, self-monitoring of blood glucose and adherence to medicines. Implementation of more than one self-care intervention found to reduce the overall healthcare cost of diabetic patients compared to patients relying on only one of the latter components. Impact of diabetic self-care intervention by pharmacist found to significantly reduce HbA1C levels of diabetic patients along with reduction of yearly healthcare cost by $1031 per beneficiary showing the effectiveness of intervention. Pharmaceutical care intervention with involvement of pharmacists proven to further improve in patient's quality of life with the spending cost of $88,600 per QALYs gained compared to higher cost in usual care. This review paper showed that pharmacist involvement in diabetic self-care interventions are proven to be cost effective and can significantly affect condition of the diabetic patients and reduce the risk of complications.

## Author contributions

All authors listed have made a substantial, direct and intellectual contribution to the work, and approved it for publication.

### Conflict of interest statement

The authors declare that the research was conducted in the absence of any commercial or financial relationships that could be construed as a potential conflict of interest.
